# Chronic Leptomeningitis and Spinal Intradural Mass Secondary to* Alternaria* Infection in a Patient with Ventriculoperitoneal Shunt

**DOI:** 10.1155/2016/4693409

**Published:** 2016-10-20

**Authors:** Sanjeet S. Dadwal, Russell Thompson, Rahul Jandial, Bernard Tegtmeier, Mike Yue Chen

**Affiliations:** ^1^Division of Infectious Disease, City of Hope National Medical Center, Duarte, CA, USA; ^2^Washington University School of Medicine, St. Louis, MO, USA; ^3^Division of Neurosurgery, City of Hope National Medical Center, Duarte, CA, USA

## Abstract

Fungal infection following placement of ventriculostomy or ventriculoperitoneal (VP) shunt is uncommon. We report the first case of* Alternaria* related central nervous system (CNS) shunt infection in a patient with CNS ependymoma manifesting as leptomeningitis and a spinal intradural mass. This case illustrates the diagnostic and management challenges.

## 1. Introduction

Infection is a major concern following placement of ventriculostomy or ventriculoperitoneal (VP) shunt with rates reported between 0 and 20%. The infection may result from contamination of the device during insertion or during routine care/manipulation and colonization at the insertion site from skin flora [1]. Central nervous system (CNS) fungal infections related to VP shunts are uncommon [2–4] and pose significant diagnostic and management challenges [1, 4]. The CNS fungal infections may manifest acutely with dire consequences especially in those with altered immune function such as those in hematologic malignancies and transplant recipients. However, in other patient populations the symptoms may be chronic and protean yet cause significant debilitation.

## 2. Case Report

A 48-year-old male presented in 9/2006 at an outside hospital with headaches. Magnetic resonance imaging (MRI) of the brain revealed a posterior fossa mass. He had craniotomy with biopsy of the mass and histopathology was consistent with grade II ependymoma. The postoperative course was complicated by obstructive hydrocephalus that required ventriculostomy and one month later an occipital VP shunt was placed. Cranial radiation therapy was administered totaling 54 Grays in 30 fractions to the field surrounding the tumor bed in 10/2006. He did well for a year until he developed worsening back pain and restaging MRI revealed diffuse leptomeningeal involvement of the spine. He was presumed to have drop metastasis and underwent whole spine radiation with a total dose of 41 Gray in 11/2007. The brain MRI over the following year revealed slow progression with extensive leptomeningeal enhancement involving the perimesencephalic cistern, bilateral foramina of Luschka, bilateral internal auditory canals, interpeduncular cistern, prepontine cisterns, quadrigeminal cistern, and right tentorium. MRI of the spine demonstrated leptomeningeal enhancement of the cervicothoracic and distal spinal cord with a large lumbosacral intrathecal mass consistent with residual ependymoma or postradiation scar. He was started on temozolomide in 5/2009.

**Figure 1:**
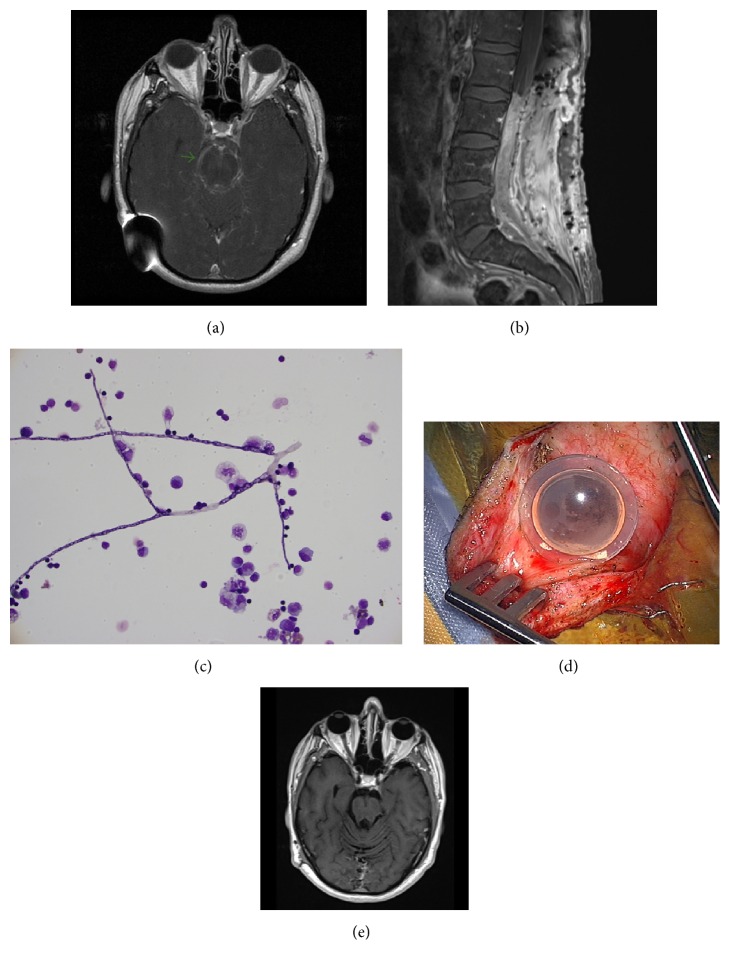
((a) & (b)) Pretreatment MRI images, (c) CSF cytology showing hyphae and inflammatory response, (d) discolored Ommaya reservoir, and (e) posttreatment MRI.

In June 2009, patient transferred his care to our institution with the diagnosis of diffuse cranial and spinal leptomeningeal ependymoma with lumbosacral drop metastasis. He was symptomatic with numbness in his right leg and back pain. He denied headaches, neck stiffness, visual changes, fever, or chills. He was continued on temozolomide. A spinal tap in 9/2009 showed cerebral spinal fluid (CSF) pleocytosis (WBC 436, neutrophils 59%, lymphocytes 17%, monocytes 12%, and unclassified cells 12%), low glucose (39 mg/dL), and high protein (636 mg/dL). Cytology and cultures (bacterial, fungal, and acid fast bacilli) were negative. He completed 4 cycles of temozolomide in 12/2009 and underwent radiation (17 Gy) to the lumbosacral spine in 1/2010. He was started on carboplatin and VP-16 in 1/2010 for progression of leptomeningeal disease and completed 8 cycles in 8/2010. A VP shunt revision was performed in 4/2010 along with multiple sessions of intracranial shunt programming for management of hydrocephalus. Between May and July 2010 he started to complain of lower extremity weakness, urinary incontinence, and developed foot drop. In 9/2010, MRI of the brain revealed stable leptomeningeal enhancement surrounding the pons, midbrain, and quadrigeminal cistern. MRI of the spine showed persistent enhancement of the cervical, thoracic, and lumbar spine and a 9 cm long enhancing soft tissue mass filling the thecal sac from L3 to S1 (Figures 1(a) and 1(b)).

In September 2010, he had spinal decompressive surgery for management of L3 to S1 intradural mass that was causing symptoms of right leg weakness, urinary incontinence, and back pain. Intraoperatively, a dense mass consisting of an inseparable tangle of nerve roots and scant fibrous tissue was identified. Surprisingly, histopathology displayed necrotic tissue with severe acute and chronic inflammation with single septate hyphae. Culture of the surgical tissue was not sent. The serum* Aspergillus* galactomannan antigen, 1,3-beta D-glucan,* Coccidioides immitis* serology,* Histoplasma* antigen, and* Blastomyces* serology were negative. CSF obtained in the first week of October 2010 was as follows: WBC 179, RBC 3, neutrophils 78%, lymphocytes 10%, monocytes 12%, glucose 73 mg/dL, and protein 125 mg/dL. The CSF* Aspergillus* galactomannan antigen, cryptococcal antigen, and* Coccidioides immitis* serology were negative. The bacterial, AFB, and fungal cultures were negative. No occult site of fungal infection was identified on CT scan of sinus, chest, and abdomen. Voriconazole was initiated in 10/2010 at therapeutic doses based on body weight. MRI of the brain and spine was repeated a month later that depicted improvement in the leptomeningeal enhancement with the exception of the L3-S1 intradural mass, which remained unchanged. By December 2010, the CSF profile improved (WBC 15, neutrophils 3%, lymphocyte 77%, monocytes 6%, protein 98 mg/dL, and glucose 46 mg/dL). MRI of the brain in 2/2011 displayed a significant decrease/resolution of abnormal leptomeningeal enhancement. MRI of the spine also showed near resolution of the cervicothoracic leptomeningeal enhancement, whereas the intradural filling defect in LS spine was unchanged. A follow-up spinal tap in February 2011 showed worsening pleocytosis (WBC 79, neutrophils 18%, lymphocytes 49%, monocytes 17%, and eosinophils 5%), increased protein (175 mg/dL), and for the first time cytology showed septate fungal hyphae with morphology suggestive of* Alternaria* (Figure 1(c)). The CSF culture eventually grew* Alternaria spp*. He was hospitalized and treated with liposomal Amphotericin B (L-Amb) and Voriconazole pending antifungal susceptibility testing at a reference lab.

All CNS hardware (VP shunt and Ommaya reservoir) was removed and an external ventricular drain (EVD) was placed. The Ommaya reservoir was discolored and a moldy black substance was seen on macroscopic exam (Figure 1(d)). L-Amb was stopped due to worsening renal function. A new VP shunt was placed for chronic obstructive hydrocephalus after repeated CSF in 3/2011 showed a decline in WBC to 7 cells and was negative for fungal growth. Based on the information from the antifungal susceptibility testing (Table 1), the patient was maintained on intravenous Voriconazole for one year from March 2011 with close monitoring of Voriconazole trough levels (the trough levels on oral formulation were subtherapeutic despite weight based dosing).

Follow-up MRI indicated no residual brain or cervicothoracic spinal leptomeningeal enhancement (Figure 1(e)). The L3-S1 spine intradural soft tissue mass has remained unchanged. CSF analysis from March 2012 showed 2 WBC, 2 RBC, mildly elevated protein, and normal glucose. He was noted to have improvement in lower extremity weakness; however, the foot drop persisted. Most recent MRI of the brain and spine shows no interval change.

## 3. Discussion

*Alternaria spp. *are dematiaceous hyphomycetes with worldwide distribution and are commonly found in soil, wood, and decomposing plant debris [5]. They are also found on intact human skin and conjunctiva [6].* Alternaria *is an emerging opportunistic pathogen. It has been implicated in allergic rhinosinusitis, asthma, onychomycosis, ocular infections especially in the setting of contact lens use/ophthalmologic surgery, cutaneous/subcutaneous infections, especially the solid organ transplant recipients, and, lastly, invasive disease in patients with hematologic malignancy [6–9]. Only one case of fatal* Alternaria* primary brain abscess has been reported in a child with chronic granulomatous disease [10].

Central nervous system device related fungal infections are an uncommon occurrence [2, 3] with the most commonly reported isolate being* Candida spp. *[3, 11]. These infections can be difficult to diagnose and often pose management challenges. The difficulty in diagnosis may result from the low yield of culture, low index of suspicion for fungal infection, and the lack of typical symptoms such as fever, headaches, neck pain, or nuchal rigidity.

In this report, we have described the clinical course of an occult fungal leptomeningeal infection caused by* Alternaria spp*., in a patient with a CNS malignancy that required multiple surgical interventions for the management of obstructive hydrocephalus. This created the setting for CNS device related infection, as higher infection rates are reported with secondary shunt surgery [12]. In our patient it was very difficult to determine the exact time of onset of infection because typical symptoms or signs of meningitis were lacking and the findings noted on MRI were considered as malignancy related. CSF pleocytosis, elevated protein level, and low glucose on initial CSF suggested an inflammatory process; however, microbiologic work-up was negative which suggested possibility of partially treated malignancy.

The diagnosis of CNS fungal infection can be particularly challenging as demonstrated by this case. The first sign of fungal infection was the histologic observation of solitary hyphae with acute and chronic inflammation in the intradural mass. Ultimately, CSF from the Ommaya reservoir grew* Alternaria spp*. and concurrently had an elevated CSF eosinophil count. Although* Alternaria* has never been reported as the cause of chronic leptomeningitis, other dematiaceous fungi such as* Scedosporium*,* Cladophialophora*, and* Wangiella* have caused devastating CNS infections in both immunocompetent and immunocompromised hosts. Dimorphic fungi such as* Histoplasma* and* Coccidioides* have been described as a cause of chronic meningitis. Hagman et al. described case of CNS* Coccidioides immitis* infection manifesting as “hyphal form” in the brain parenchyma and CSF, especially in the setting of a VP shunt or Rickham reservoir [13].

The management of infected CNS shunts is challenging. It involves appropriate antimicrobial therapy (with reliable penetration into the CSF and brain tissue) targeting the identified pathogen, removal of the infected CNS shunt, and resection of infected tissue, which is not always feasible. In our patient, the initial antifungal agent, Voriconazole, was chosen based on the histological evidence of “septate hyphae” that suggested the possibility of* Aspergillus*, although non-*Aspergillus* like molds were in the differential diagnosis. Voriconazole has good in vitro activity against* Alternaria spp*., and the isolate was sensitive based on MIC breakpoint (Table 1).

Long-term Voriconazole use can be complicated with problems such as drug related hepatotoxicity and phototoxicity with risk for skin cancer. Our patient exhibited both of these side effects with the exception of skin cancer. We also encountered challenge in maintaining adequate trough level with oral formulation that was overcome by intravenous dosing. In vitro, Amphotericin B and Posaconazole have activity against this pathogen and can be used as second-line agents. Amphotericin B lipid formulations can lead to nephrotoxicity, which our patient developed. There is a paucity of data and recommendations regarding the duration of antifungal therapy in such situations. We treated our patient for one year with intravenous Voriconazole at therapeutic doses (weight based). Repeated CSF analyses and MRI of the brain/spine were done to measure treatment response. At the one year treatment mark, CSF pleocytosis had resolved and MRI indicated resolution of the brain/cervicothoracic leptomeningeal inflammation. However, the lumbar intradural spine mass remained unchanged. Clinically, the patient has regained functional capacity, although lower extremity neurological problems persist.

## 4. Conclusion

This case description emphasizes the challenges faced in establishing the diagnosis of a CNS device related fungal infection and its management in the setting of a CNS malignancy.

## Figures and Tables

**Table 1:** Antifungal susceptibility.

Antifungal agent	MIC (mg/mL)
Amphotericin B	0.54
Itraconazole	0.5
Posaconazole	0.25
Voriconazole	1.00
